# Myocardial Deformation by Echocardiogram after Transcatheter Aortic Valve Implantation

**DOI:** 10.5935/abc.20170013

**Published:** 2017-05

**Authors:** Carolina Stangenhaus, Marcelo Luiz Campos Vieira, Claudio Henrique Fischer, Antonio Carlos Bacelar Nunes Filho, Marco Antonio Perin, Adriano Mendes Caixeta

**Affiliations:** Hospital Israelita Albert Einstein, São Paulo, SP - Brazil

**Keywords:** Myocardium/physiopathology, Echocardiography. Doppler, Transcatheter Aortic Valve Replacement, Aortic Valve Stenosis

## Introduction

Myocardial deformation (strain) analysis may be done through echocardiography from data obtained by tissue doppler technique or two-dimensional images from speckle tracking, allowing the calculation of longitudinal, circumferential and radial deformations of myocardial fibers.^1,2^ Myocardial strain analysis has recently been used in the evaluation of regional myocardial movement and function and in the systolic time-to-peak calculation which studies cardiac synchronicity and myocardial electromechanical coupling.^1^ In clinical practice, conventional echocardiographic investigation is helpful in detecting global myocardial dysfunction and alterations in ventricular segmental contractility. In cardiotoxicity from the use of chemotherapeutics, we may find alterations in cardiac mechanics with no modification of the left ventricle ejection fraction (LVEF). Thus, the analysis of myocardial deformation brings relevant information in the analysis of regional ventricular systolic dysfunction in subclinical conditions.^2^

Similarly, previous investigations have shown that the study of myocardial deformation is able to detect sudden and early alterations in systolic function of valve disease patients, even before they present LVEF modifications.^1,3,4^ This phenomenon is due to a significant decrease in myocardial deformation in three different spatial planes, triggering deformation modifications in the longitudinal, circumferential and radial axes.^5^ In patients who present reduced longitudinal myocardial deformation, we can observe radial deformation that is superior to normality parameters, which may result in preserved ventricular function, when estimated by LVEF. Other applications of myocardial deformation include amyloidosis, hypertrophic cardiomyopathy, right ventricular dysfunction, athlete's heart, cardiac dyssynchrony and valve diseases (mitral insufficiency and aortic stenosis).^2^

Transcatheter aortic valve implantation (TAVI) appeared as a new option for the treatment of inoperable aortic stenosis or high surgical risk patients.^6^ The use of myocardial deformation with echocardiography in the immediate evaluation of aortic stenosis patients undergoing TAVE is underexplored.

### Case 1

Patient, female, 79 years old, with atrial fibrillation and pulmonary hypertension, and decreased functional capacity in the last year. Three-dimensional transesophageal echocardiography showed presence of aortic valve stenosis with maximum transvalvular gradient of 67 mmHg and mean of 39 mmHg, valve area of 0.5 cm^2^, and LVEF (Simpson's method) of 60%. Pre-TAVI strain analysis was -14% and after implantation of aortic prosthesis Sapien XT of 23 mm (Edwards Lifescience, USA) immediate improvement of the myocardial deformation to -20% was observed.

### Case 2

Patient, male, 81 years old, with cirrhosis from hepatitis B and hypothyroidism, symptomatic, functional class II (NYHA) from aortic valve stenosis. Implantation of aortic valve Sapien XT of 26 mm, with immediate improvement of the strain from -15% to -22%. Myocardial dyssynchrony evaluation with analysis through pre TAVI systolic time-to-peak calculation method went from 132 ms to 65 ms after the procedure.

### Case 3

**Figure 1:**
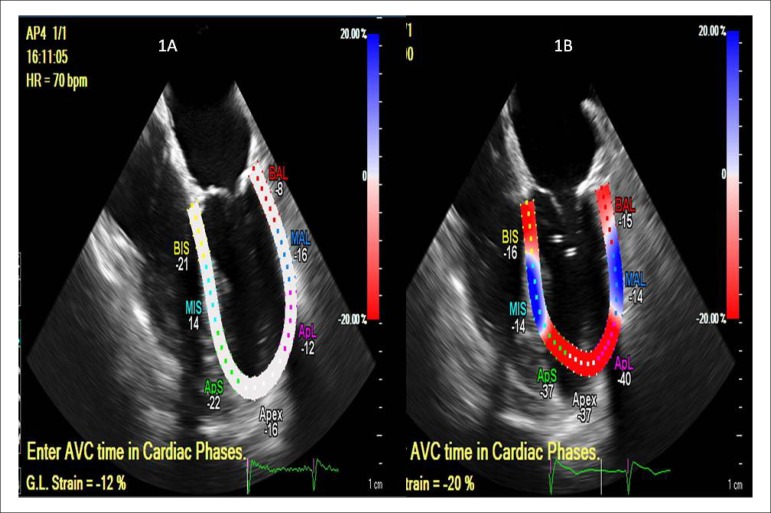
Global longitudinal strain before (Figure 1A: -12%, VN < -20%) and after (Figure 1B: -20%) percutaneous implantation of aortic prosthesis. Case 3 patient.

Patient, male, 77 years old, with aortic stenosis for 10 years, with recent worsening of functional class. Echocardiogram showed maximum transvalvular gradient of 87 mmHg and mean of 49 mmHg, valve area of 0.7 cm^2^ and LVEF of 57%. Implantation of aortic valve Sapien XT, 23 mm, with immediate normalization of the pre-procedure myocardial strain from -12% to -20% after the procedure (Figure 1).

### Case 4

**Figure 2:**
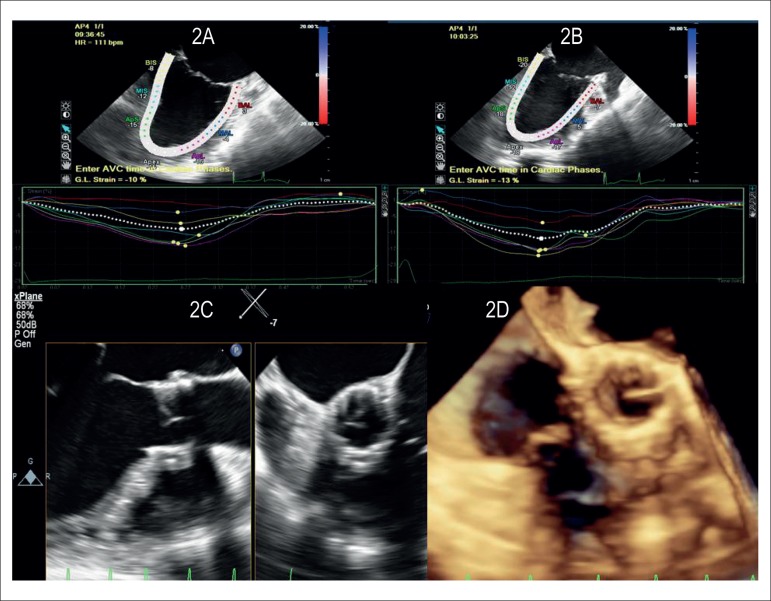
Myocardial strain analysis before (Figure 2A : -10%) and after (Figure 2B: -13%) percutaneous implantation of aortic prosthesis. Figure 2C: demonstration with two-dimensional transesophageal echocardiography of implanted aortic prosthesis, left figure (longitudinal plane), right figure (transverse plane). Figure 2D: demonstration with three-dimensional transesophageal echocardiograph of the implanted aortic prosthesis, en face. Case 4 patient.

Patient, female, 74 years old, with aortic stenosis for 20 years, functional class II (NYHA). Echocardiogram showed maximum transvalvular gradient of 65 mmHg and mean of 38 mmHg, valve area of 0.6 cm^2^ and LVEF of 28%. Implantation of aortic valve Sapiens XT, 23 mm, with immediate improvement of the myocardial strain from -10% to -13% and LVEF improvement to 34% (Figure 2). 

## Discussion

Aortic valve stenosis evolution results in pressure overload in the myocardial wall, leading to increased cardiac thickness. Ventricular hypertrophy progresses as aortic stenosis becomes more significant, an adaptation mechanism for ventricular function maintenance. However, this compensatory mechanism is finite, triggers an increase in left ventricular volume (positive remodeling) and a decrease in ejection fraction over time.^4^

Lancellotti et al.,^5^ in studies using echocardiography, observed the following alterations in patients with aortic stenosis:

1. asymptomatic patient with significant aortic stenosis presents expressive increase in global left ventricular afterload;
2. Ventricular afterload increase negatively affects myocardial function, especially in the axial deformation, despite the normal ejection fraction;
3. Elevated afterload in low-flow aortic stenosis patients, when systemic arterial compliance is reduced;
4. The state of low-flow is related to a compromised diastolic function and reduction of myocardial deformation. Longitudinal, radial and circumferential myocardial deformations are significantly compromised in patients with elevated afterload. In an initial stage of aortic valve stenosis, however, there is only a reduction in the longitudinal myocardial deformation, mainly related to alterations in subendocardial cardiac fibers. Radial and circumferential deformations may be, paradoxically, vicarious in this period. Subsequently, with the progression of aortic stenosis severity, we now have a reduction of the radial and circumferential deformation due to modifications of the fibers of the mid-myocardial layer which present circumferential spatial alignment.

Current guidelines recommend aortic valve replacement in symptomatic or asymptomatic aortic valve stenosis patients with reduction of the ejection fraction.^6,7^ When compared to patients with preserved systolic function, those with reduced ejection fraction present less favorable clinical evolution after valve repair.^4^ In patients with preserved ventricular function, myocardial deformation analysis allows early detection of subclinical alterations of myocardial mechanics, which could, after the procedure, positively result in longer survival and better quality of life. 

In a study by Delgado et al.,^4^ aortic valve replacement surgery improved the analysed parameters through speckle tracking, even though the ejection fraction remained unaltered. Additionally, these authors report an improvement in myocardial deformation after valve replacement. That solidifies the ability of myocardial deformation analysis to detect sudden alterations in systolic function in patients with severe aortic stenosis. 

In another study, Bauer et al.,^3^ who assessed 8 patients submitted to TAVI, showed improvement in global and regional systolic function of the left ventricle, even in patients with reduced ejection fraction.

Similarly, Sebastian et al.^6^ observed an improvement of myocardial mechanics in patients submitted to TAVI 12 months after the procedure. Becker et al.^7^ showed a positive response in myocardial deformation 7 days and 6 months after TAVI. Specifically, there was an improvement in the ejection fraction of 51±6% pre-TAVI to 54±4% and 57±3%, 7 days and 6 months after the procedure. For circumferential myocardial deformation analysis, there was an improvement of -14.9±1 pre-TAVI to -16.1±1.2 and -17.3±1.5 in 7 days and 6 months after TAVI, respectively. 

In a similar way, in the present 4-case report, we observed significant improvement of myocardial deformation analysis immediately after TAVI. Three patients presented preserved LVEF, and one (Case 4), presented significant ventricular dysfunction, with improvements observed in LVEF and longitudinal myocardial deformation, immediately after the procedure. These data confirm that left ventricular function in valvulopathies directly depends on the afterload, and immediate relief of this variable may positively influence clinical outcomes. The causal relation between immediate recovery of myocardial deformation after TAVI - even in patients with preserved left ventricular function - and the benefits in symptoms and morbidity and mortality should be explored in future studies.
